# Effect of rest duration between sets on fatigue and recovery after short intense plyometric exercise

**DOI:** 10.1038/s41598-024-66146-2

**Published:** 2024-07-02

**Authors:** Michał Staniszewski, Joanna Tkaczyk, Anna Kęska, Przemysław Zybko, Anna Mróz

**Affiliations:** 1 Department of Water and Winter Sports, Józef Piłsudski University of Physical Education in Warsaw, Warsaw, Poland; 2 Department of Human Biology, Józef Piłsudski University of Physical Education in Warsaw, Warsaw, Poland; 3 Department of Biomedical Sciences, Józef Piłsudski University of Physical Education in Warsaw, Warsaw, Poland

**Keywords:** Muscle torques, Lactate, Ammonia, Peak power, Biochemistry, Bone quality and biomechanics, Skeletal muscle, Musculoskeletal system

## Abstract

Plyometric training is characterized by high-intensity exercise which is performed in short term efforts divided into sets. The purpose of the present study was twofold: first, to investigate the effects of three distinct plyometric exercise protocols, each with varying work-to-rest ratios, on muscle fatigue and recovery using an incline-plane training machine; and second, to assess the relationship between changes in lower limb muscle strength and power and the biochemical response to the three exercise variants employed. Forty-five adult males were randomly divided into 3 groups (n = 15) performing an exercise of 60 rebounds on an incline-plane training machine. The G0 group performed continuous exercise, while the G45 and G90 groups completed 4 sets of 15 repetitions, each set lasting 45 s with 45 s rest in G45 (work-to-rest ratio of 1:1) and 90 s rest in G90 (1:2 ratio). Changes in muscle torques of knee extensors and flexors, as well as blood lactate (LA) and ammonia levels, were assessed before and every 5 min for 30 min after completing the workout. The results showed significantly higher (*p* < 0.001) average power across all jumps generated during intermittent compared to continuous exercise. The greatest decrease in knee extensor strength immediately post-exercise was recorded in group G0 and the least in G90. The post-exercise time course of LA changes followed a similar pattern in all groups, while the longer the interval between sets, the faster LA returned to baseline. Intermittent exercise had a more favourable effect on muscle energy metabolism and recovery than continuous exercise, and the work-to-rest ratio of 1:2 in plyometric exercises was sufficient rest time to allow the continuation of exercise in subsequent sets at similar intensity.

## Introduction

Plyometric training is characterized by muscle action in a stretch–shortening cycle (SSC). There are various proposed mechanisms to explain the SSC phenomenon found in the literature. Researchers have highlighted the importance of elastic strain energy, involuntary nervous processes, increased active range of movement, length-tension characteristics, preactivity tension, and improved coordination due to the innate action of the prestretch^1^. Pre-stretching the stimulated muscles significantly increases movement efficiency because potential elastic energy is stored in the muscle–tendon complex and then returned during muscle contraction, increasing its speed, strength, and power compared to muscle contraction without a pre-stretch. It has been shown that training using a rapid transition from eccentric to concentric muscle work is very effective in developing motor fitness, especially muscle strength, as it creates both the muscular and nervous systems^2–5^.

Continuous training methods are characterized by the absence of rest intervals during exercise and a large volume of exercise performed mainly under conditions of oxygen balance. The intermittent method involves alternating between load and rest phases. The purpose of rest intervals between exercise sets is to allow recovery and to perform the next set at a similar intensity^6–8^. Intermittent methods are based on reports indicating that the use of rest intervals significantly influences metabolic processes during muscle work, neuromuscular coordination, the magnitude of the tolerable load in subsequent exercise sets, and the development of fatigue^9–11^. However, the effectiveness of intermittent methods is largely determined by the rest interval duration, which should be as precisely planned as the exercise itself to ensure adequate recovery effect^12^. According to the American College of Sports Medicine^13^, the duration of rest intervals between successive sets of intermittent exercise should be between 30 s and 5 min, depending on the planned training goal. However, it is assumed that in short intense exercises, the duration of the rest intervals between sets should depend on the duration of exercise in the sets and is usually determined by a work-to-rest ratio of 1:1 to 1:3. This means that if, for example, it takes about 45 s to 1 min to perform an intense exercise sets of 15–20 repetitions, the rest duration between such sets should be 45 s to 3 min^14–16^.

Muscle fatigue is the inability of a muscle or group of muscles to produce the required static force or mechanical power within a set time, or the inability to perform the total amount of work under certain conditions^17^. The development of muscle fatigue is determined among others by the availability of energy substrates and the accumulation of products of their metabolism^18–20^. Muscle work requires the intensification of various processes in muscle cells to meet the high demand for ATP. One of them, anaerobic metabolism, reaches maximum efficiency at the beginning of the exercise, and, by providing large amounts of ATP in a short time, it enables the performing of high-intensity exercise. In contrast, aerobic metabolism determines the ability to perform an exercise of longer duration, during which, however, lower power output (1/3 of anaerobic power) is achieved^21,22^. It follows, therefore, that the maximum strength capacity of muscles, determined by efficient ATP resynthesis, decreases as intense exercise continues, and that fatigue builds up from the beginning of exercise^23^.

During high-intensity intermittent exercise, the main processes of ATP resynthesis are phosphocreatine breakdown and anaerobic glycolysis, leading to the production of lactate^24,25^. An increase in exercise intensity is associated with the production of lactate which contributes to the development of fatigue^26,27^. Thus, in the case of intermittent exercise, the ability to continue exercise through subsequent exercise sets largely depends on the efficiency of phosphocreatine resynthesis and lactate removal, processes that occur during rest intervals^28^.

The breakdown of ATP during exercise generates the formation of AMP, whose deamination leads to an increase in ammonia in working muscles. Its source can also be amino acids derived from muscle proteins^29^. Physical effort accelerates protein turnover, and the contribution of amino acids (mainly branched-chain amino acids) to ATP production increases, especially when carbohydrate availability is low^30^. It is worth noting that during short-term, high-intensity exercise, ammonia mainly comes from AMP deamination^31^. Manipulating the work-to-rest ratio in intermittent plyometric exercises can significantly impact performance, maximal force output, and metabolic variables. Optimal ratios are often tailored to specific training goals, with shorter rest intervals promoting greater metabolic stress and endurance adaptations, while longer rest intervals allow for maximal force regeneration and power output. Finding the right balance in the work-to-rest ratio is crucial for designing plyometric training programs that effectively target the desired physiological adaptations and enhance overall athletic performance. The present study intended to examine how the rest interval duration between sets or lack of rest intervals affects the ability to continue short intense lower limb plyometric exercise. The main aim of the study was to compare the effects of different work-to-rest ratios on muscle fatigue and recovery in and after such effort and to assess the relationship between changes in lower limb muscle strength and power and the biochemical response to the three exercise variants used.

## Results

### Workout

**Figure 1:**
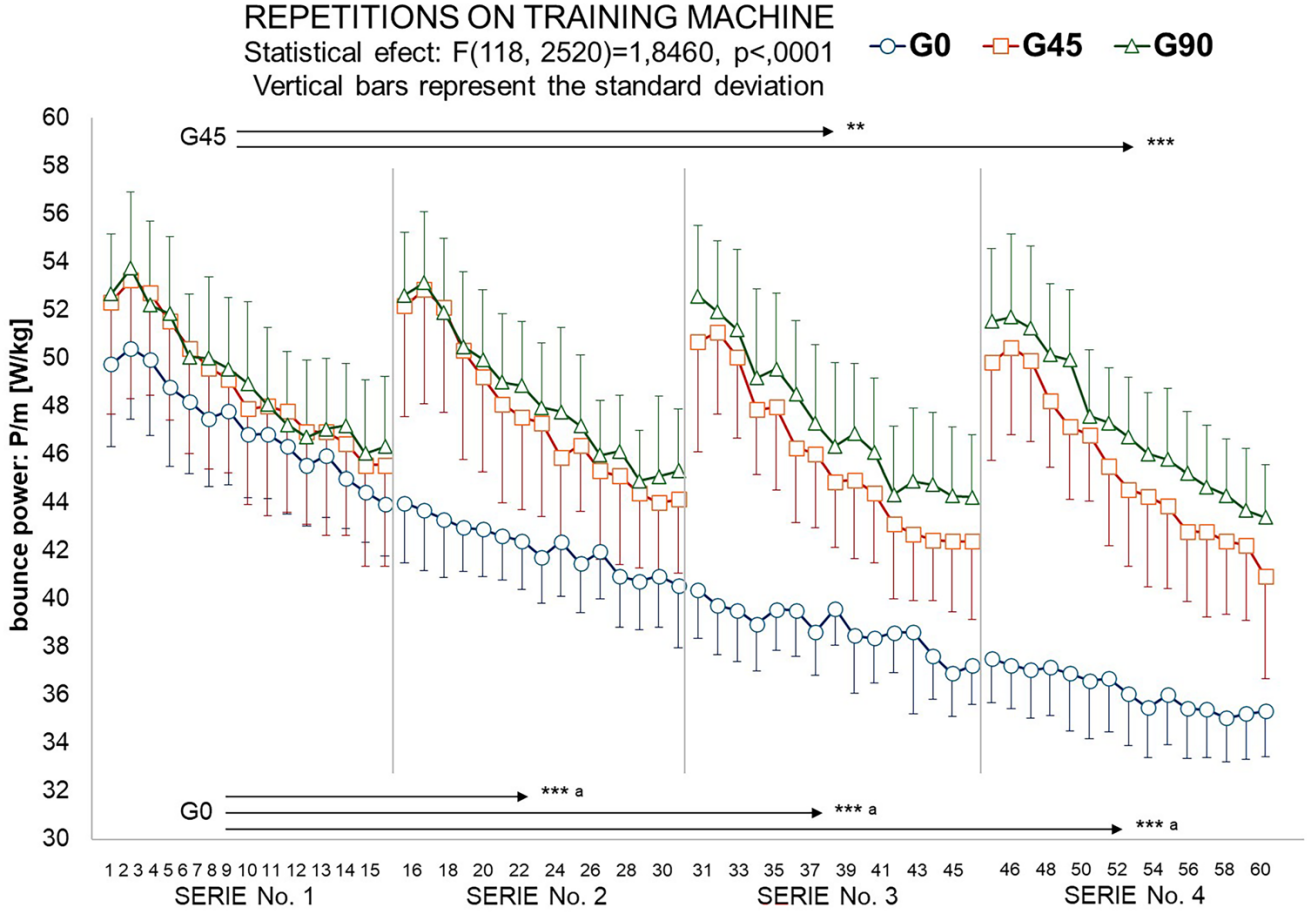
Characteristics of the mean peak power generated in successive sets of exercise relative to body mass (W/kg). The arrows indicate in which subsequent sets of exercise the power values were significantly lower than in the first one. **p < 0.01 ***p < 0.001—values significantly lower than in the first set. ^a^*p* < 0.05—values significantly lower than in G45 and G90.

Figure 1 shows the group-averaged power of each repetition relative to body weight. Due to the continuous character of workout in the G0 group, a linear decrease in peak power output as a function of exercise duration is observed. The G45 and G90 group had rest intervals following each 15 repetitions allowing them to start the next set at a similar power level. The mathematical division of 60 repetitions of continuous exercise into 4 equal parts (4 sets), performed after data collection, allowed intergroup comparison of mean power obtained in successive sets. ANOVA showed significant between-group differences in the mean power for the entire exercise (F_2,42_ = 7.75, *p* = 0.001, η^2^ = 0.270) and statistically significant differences in power in successive sets and between groups (F_6,126_ = 13.54, *p* < 0.001, η^2^ = 0.392). No statistically significant differences were found between the total mechanical work performed in each group. Based on post hoc tests, it was shown that the values of repetitions 1–15 and the mean power of the first set were not statistically significantly different between the groups (*p* > 0.05). In next series the G0 mean power was significantly lower than in both G45 and G90 groups (lower by13–15% in 2nd, 18–23% in 3rd and 25–30% in 4th set). Each of the successive 15 repetitions in the G0 group was characterized by significantly lower power than the preceding set. In sets 3 and 4, G45 participants generated significantly lower relative power than in set 1. In the group with the longest intervals (G90), there were no significant differences in power between sets.

### Muscle torques

**Figure 2:**
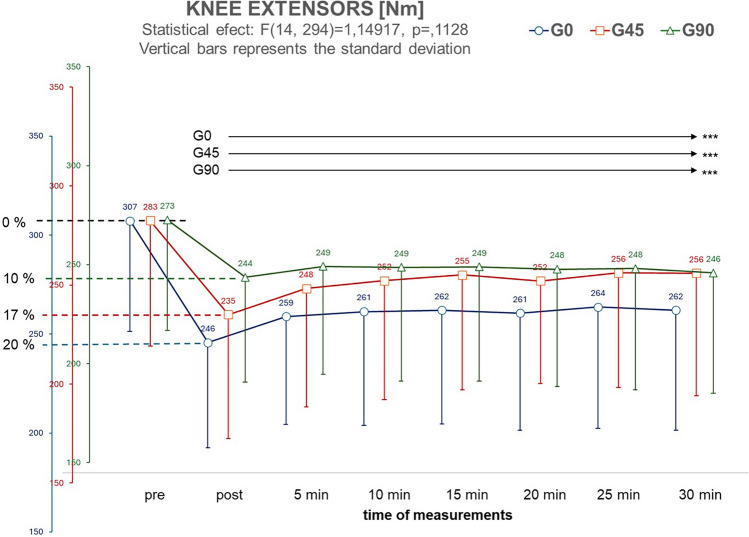
Changes in knee extensors muscle torques (Nm) before exercise (pre) and in the successive 7 measurements taken every 5 min after exercise. The arrows indicate the range of measurements in which muscle torque values remained significantly lower than pre-exercise (***p < 0.001).

The parameter that monitored the post-exercise biomechanical fatigue was the measurements of the muscle torques in the knee extensors (KE) and flexors (KF), which were performed for 30 min following the exercise. ANOVA revealed that the mean values of muscle torques at baseline were not statistically significantly different between groups (KE: F_2.42_ = 0.997, *p* = 0.337, η^2^ = 0.045; KF: F_2.42_ = 1.122, *p* = 0.335, η^2^ = 0.051). Immediately after exercise, the values of KE muscle torques (Fig. 2) were significantly lower in all groups than before exercise (F_2.42_ = 3.483, *p* = 0. 039, η^2^ = 0.142). The largest difference was found in the G0 group (Δ = − 20%), and the smallest in the G90 group (Δ = − 10%). The recorded KE muscle torque values exhibited their lowest levels immediately post-exercise, with subsequent increments observed over the next 30 min. However, the variations in these increments were highly individualized, prevented the determination of consistent patterns or general characteristics across the groups and made ANOVA characteristic Measurements × Group not significant (F_14.294_ = 1.4917, *p* = 0.113, η^2^ = 0.066). The nuanced and divergent responses highlight the complex and individual nature of the recovery process in muscle force and torque following exercise. None of the groups showed muscle torques in the knee extensors returning to their pre-exercise levels within 30 min of recovery.

No significant differences for Measurement × Group (F_14,294_ = 0.950, *p* = 0.505, η^2^ = 0.043) for KF muscle torques were noted. The values obtained immediately after exercise and the values from the recovery period were not significantly different from the baseline levels.

Pearson linear correlation analysis indicated a significant connection for all participants between the body mass and the values of power generated during exercise on an inclined plane (r = 0.6387, *p* < 0.001), and the values of muscle torques in static conditions (r = 0.5836, *p* < 0.001). High correlations were also found between power generated during exercise and muscle torques of KE (r = 0.5355, *p* < 0.001) and KF (r = 0.4298, *p* = 0.003).

### Blood lactate

**Figure 3:**
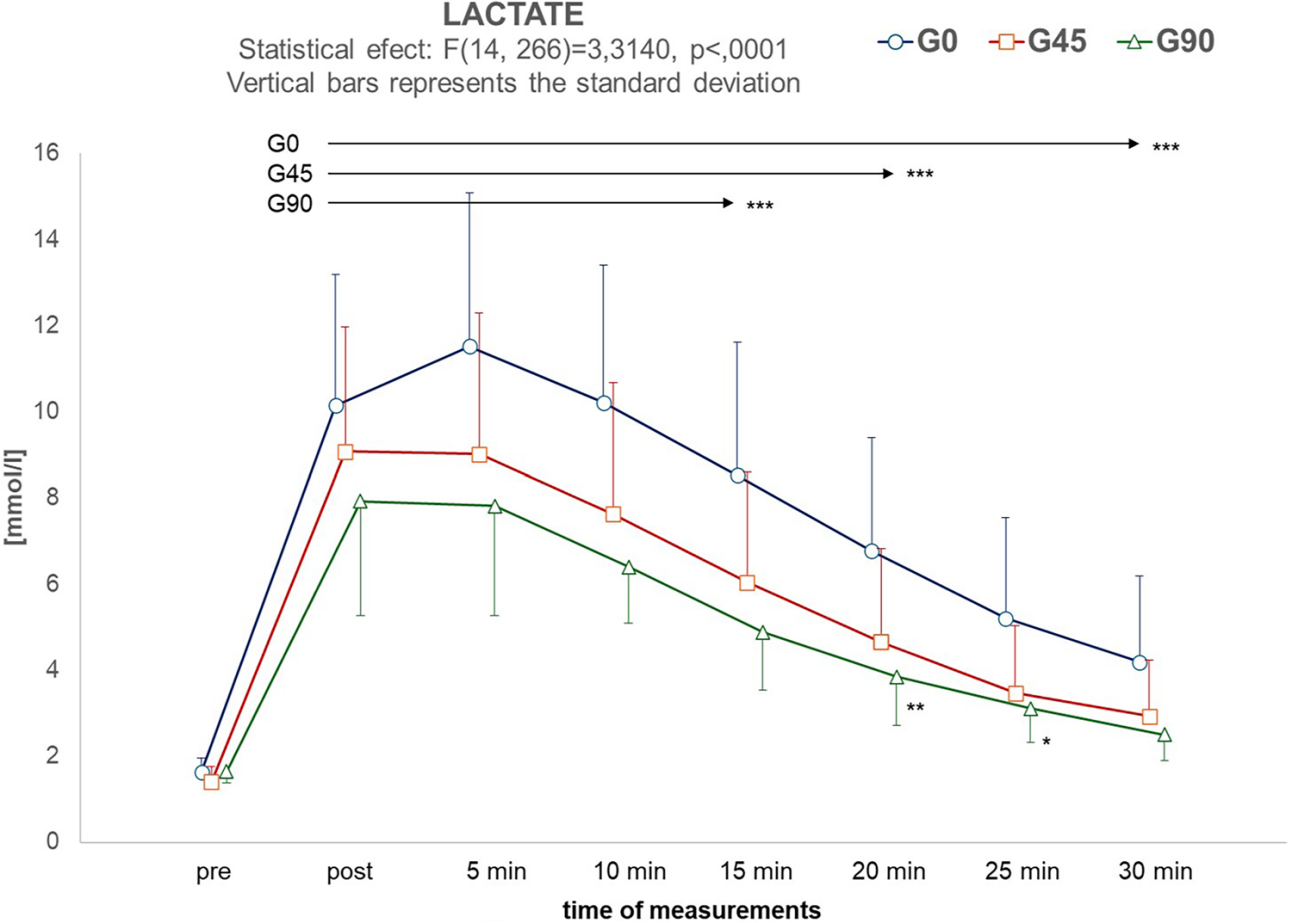
Changes in lactate levels (mmol/l) before exercise (pre) and in the successive 7 measurements taken every 5 min after exercise. The arrows indicate the range of measurements in which lactate concentration remained significantly higher (*p* < 0.001) than pre-exercise. *p < 0.05, **p < 0.01, ***p < 0.001—values significantly higher than pre-exercise.

Blood lactate (LA) level was analyzed during the 30-min recovery period after the exercise (Fig. 3). Changes in LA levels at successive measurement points followed a similar pattern in all groups, while ANOVA showed statistically significant differences between the magnitude of changes in continuous and intermittent exercise (F_2,38_ = 6.47, *p* = 0.004, η^2^ = 0.254) and for Measurement × Group (F_14,266_ = 3.31, *p* < 0.001, η^2^ = 0.148). Baseline LA levels averaged less than 2 mmol/L and were not statistically different between groups. Post-hoc analysis showed that a period of 30 min of recovery was insufficient for LA levels to return to baseline in the group without rest intervals. When the work-to-rest ratio was set at 1:1, the time for LA levels to return to baseline was 30 min, whereas it was 25 min for the 1:2 variant.

### Ammonia

**Figure 4:**
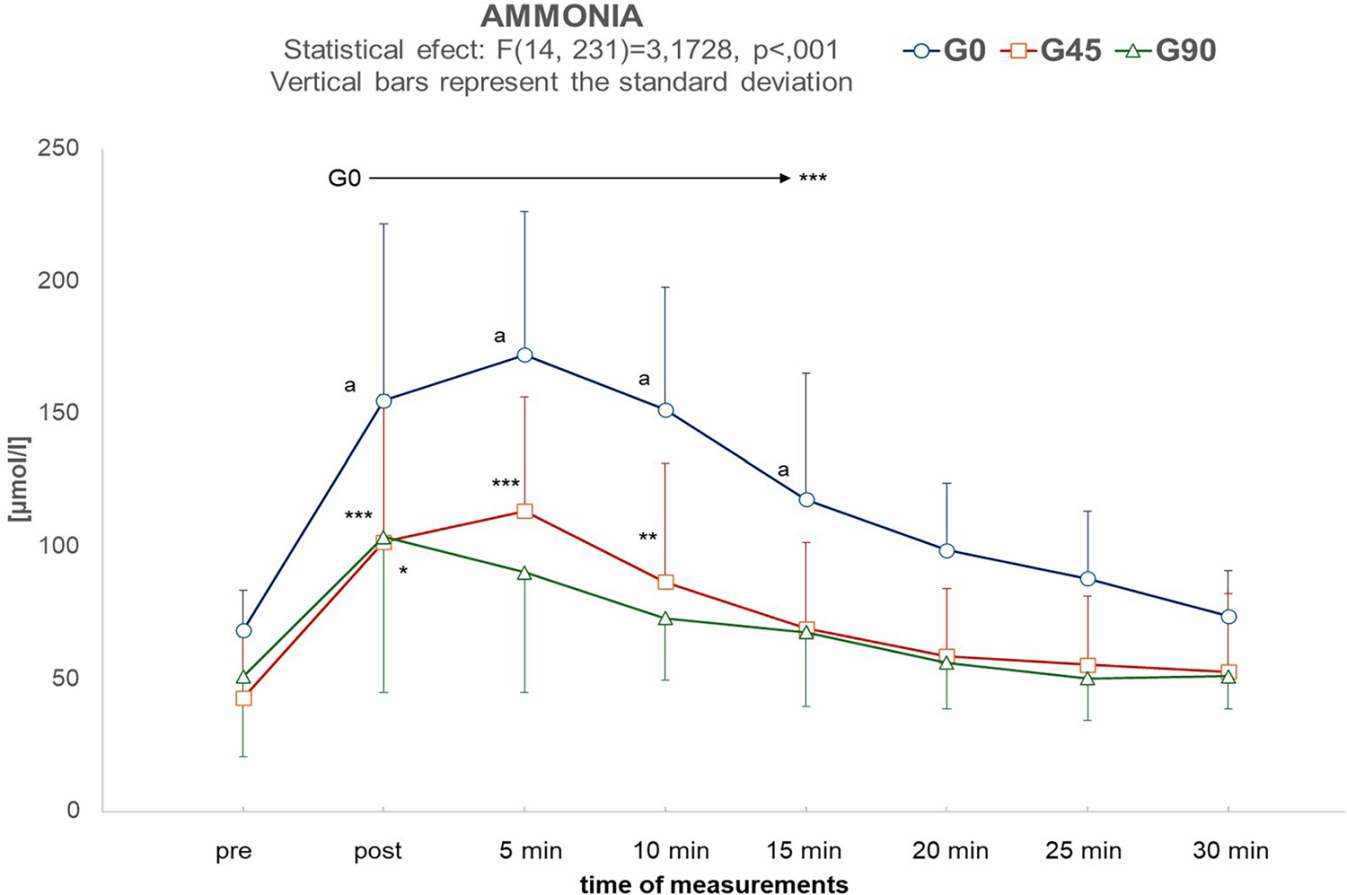
Changes in ammonia levels (μmol/l) before exercise (pre) and in the successive 7 measurements taken every 5 min after exercise. The arrows indicate the range of measurements in which ammonia concentration remained significantly higher (*p* < 0.001) than pre-exercise. *p < 0.05, **p < 0.01, ***p < 0.001—values significantly higher than pre-exercise. ^a^*p* < 0.05—values significantly higher than in groups G45 and G90.

Figure 4 shows the characteristics of changes in ammonia levels between baseline and 30 min after exercise. ANOVA showed statistically significant differences in both between-group comparisons (F_2,38_ = 15.81, *p* < 0.001, η^2^ = 0.489) and for Measurement × Group (F_14,231_ = 3.17, *p* < 0.001, η^2^ = 0.161). Post-hoc tests indicated that for the first 15 min after exercise, ammonia levels in G0 were significantly higher than in G45 and G90. A period of 20 min of recovery was sufficient for the ammonia levels in G0 to no longer be significantly higher than before training. In G45, at 15 min of recovery, ammonia concentrations were not significantly different from the baseline. In G90, ammonia levels were significantly higher than at baseline only immediately after exercise.

Statistically significant correlations were found between blood lactate and ammonia levels in individual groups: in the G0 group at 5 (r = 0.8142, *p* = 0.002), 10 (r = 0.6705, *p* = 0.024), 15 (r = 0.6858, *p* = 0.020), and 20 min (r = 0.6727, *p* = 0.023) after exercise; in the G45 group post-exercise (r = 0.8041, *p* = 0.005), and at 5 (r = 0.7611, *p* = 0.011), 10 (r = 0.8145, *p* = 0.004), 15 min (r = 0.6734, *p* = 0.033) after exercise; in the G90 group, only post-exercise (r = 0.6327, *p* = 0.037). No statistically significant correlations were found between biochemical factors and mean or maximum power of rebounds performed on the incline trainer.

## Discussion

The study was conducted to evaluate the effectiveness of using rest intervals during short intense plyometric exercise. The biomechanical and biochemical evaluation of the quality of performing exercise and the pattern of post-exercise recovery used in the study confirmed the rationality of using intervals in this type of exercise. This was evidenced primarily by the mean power obtained during exercise on the incline trainer in the studied groups. Analysis of the changes in this parameter revealed that those in the G45 and G90 groups were able, even after a short rest, to continue exercising at a significantly higher intensity compared to those performing continuous exercise (G0 group). It was further observed that between 45 and 90 s of continuous exercise, the participants performed the repetitions with an average of 11% lower power compared to the first 45 s, whereas in the last 45 s of the 3-min exercise, power was already lower by an average of 23%. In the group performing the exercise with 45-s intervals, the mean power of the first two sets remained at similar levels, while in sets 3 and 4, the participants were unable to continue the exercise at the same level (a decrease in power of about 7% on average). Only those exercising with intervals twice as long as the exercise (the G90 group) were able to perform repetitions in all sets with similar power, which leads to the conclusion that the use of the 1:2 (work-to-rest ratio) variant of plyometric training seems to provide the minimum time needed to perform the next set of repetitions with similar intensity.

Our findings are consistent with previous studies. Guan et al.^32^, for example, observed that a 60-s rest interval is sufficient between sets consisting of 10 CMJ jumps (15 s of exercise). It is worth noting that the work-to-rest ratio, in this case, was 1:4. Tredrea^33^, based on a literature review, found that in intense strength training (> 80% of 1RM), only intervals of 4–8 min allowed the next set of plyometric exercises to be performed at a similar power level. Furthermore, Lopes et al.^34^ showed that doubling the duration of the interval between sets (from 30 to 60 s) allowed participants to perform significantly more repetitions in a set but increasing the interval from 60 to 120 s no longer increased the number of repetitions.

For skeletal muscle fibres, the reduction in force generation may be due to muscle deoxygenation, resulting in a change in the nature of cellular metabolism, leading to both peripheral and central fatigue^35^. In the present study, the measurement of blood lactate and ammonia concentrations was used to assess the quality of metabolism in applied exercise and muscle fatigue. However, there was no significant relationship between the exercise load and biochemical parameters, despite the observed increase in both blood lactate and ammonia levels. However, a significant relationship was found between the power decline with the duration of continuous exercise and the blood lactic acid levels. Perhaps the lack of a direct relationship between power and biochemical parameters is due to the short duration of the exercise used, which did not lead to a significant activation of anaerobic glycolysis, as the recorded values of maximum lactate levels seem to confirm. The mean maximum lactate level did not exceed 12 mmol/l in the G0 group, 10 mmol/l in the G45 group, and 8 mmol/l in G90.

The significant post-exercise increase in blood lactate levels observed in all groups indicates that the exercise used activated anaerobic glycolysis as the main source of ATP resynthesis. Furthermore, a comparison of post-exercise lactate between the continuous and intermittent exercise groups undoubtedly shows that the use of rest intervals between exercise sets not only allows for longer exercise at a higher intensity but also shortens post-exercise recovery.

Similar to changes in lactate levels, an increase in ammonia concentration may explain the decrease in muscle biomechanical characteristics. An increase in ammonia level above 40 µmol/l can be associated with a decrease in peak power^36^. Other studies have also found that exceeding the resting ammonia concentration causes a decrease (40–50 µmol/l) in generated power, as measured by jump height^37^. Accumulation of ammonia in the blood after a high-intensity run has also been documented in recreational athletes, as well as in 400 m runners^37,38^.

The increase in ammonia levels during high-intensity exercise is mainly interpreted as an indicator of increased deamination of AMP to IMP^39^. Furthermore, ammonia levels may be an extracellular marker of muscle ATP concentration during leg press exercise^36^. Therefore, the increase in blood ammonia levels in our study indicates that the participants performed an intense exercise that required a large amount of ATP. At the same time, the observed intergroup differences seem to confirm the validity of rest intervals during lower limb plyometric exercise, allowing the resynthesis of phosphate energy sources in the muscles, which reduces exercise-induced ammonia production.

The use of simultaneous evaluation of exercise-induced changes in lactate and ammonia levels in the present study also made it possible to establish the relationships between exercise metabolites. A significant positive correlation between lactate and ammonia levels was observed in all study groups at a common point, which was the maximum ammonia level (5 min after exercise for the G0 and G45 groups and immediately after exercise for the G90 group). Thus, it can be concluded that for continuous plyometric exercise and the exercise bouts separated with rest intervals of less than 45 s, the results of blood lactate measurement may reflect the rate of AMP deamination. At the same time, the smallest increase in blood ammonia level in the group with the longest intervals between exercise sets confirms the dependence of ammonia release into blood on the increase in lactate concentration and the concomitant decline in muscle pH^40^. Given reports in the literature indicating that ammonia has a limiting effect on nervous system function and thus also impairs neuromuscular response^41^, it is advisable to use rest intervals between exercise sets in plyometric training to increase training effectiveness.

In conclusion, it can be stated that intermittent exercise had a more beneficial effect on muscle energy metabolism than continuous exercise of the same volume, and the 90 s interval after 45 s of plyometric exercise was sufficient to allow for the continuation of the exercise in subsequent sets at similar power. Post-exercise changes in the analyzed biomechanical and biochemical parameters confirm a more rapid rate of recovery after intermittent exercise. Therefore, it can be concluded that since it is expected that during lower-limb plyometric exercises, the athlete should continue successive sets at a similar intensity, intermittent training is advisable, and the duration of rest intervals should be based on a 1:2 work-to-rest ratio. However, it is worth noting that the rest intervals equal to the duration of exercise (work-to-rest ratio of 1:1) can also be used in training if it is expected to start the successive sets of exercises at incomplete muscle recovery, which may result in increased adaptation to the fatigue.

This study has a potential limitation. In further experiments, it would be worthwhile to verify whether the conclusions about the applied duration of rest intervals in plyometrics exercise are confirmed over the longer training process. We investigated only adult men, but the next studies should also include female participants. It would be advisable to perform biomechanical measurements at larger intervals (every 10–15 min) but for a longer time following exercise to assess the time for muscle torque values to return to baseline levels.

## Methods

All participants were informed about the experimental procedure. Subjects were further informed of the conditions of participation in the experiment and potential risks and received information that they could withdraw from the experiment at any time. Each person signed an informed consent to participate in the study. The experiment was approved by the University of Physical Education in Warsaw Research Bioethics Commission (SKE01-24/2020). All methods were performed in accordance with the relevant guidelines and regulations or declaration of Helsinki.

### Participants

The number of participants was based on sample size calculations using G*Power software (version 3.1.9.4, Germany. https://www.psychologie.hhu.de/arbeitsgruppen/allgemeine-psychologie-und-arbeitspsychologie/gpower). A priori sample size was calculated for a group by time interaction comparison (F test, ANOVA for repeated measures, within-between interaction) with the following specifications: alpha level = 0.05, power = 0.95, f effect size = 0.25. Based on this the estimated number of subjects was 30.

The study examined forty-five male physical education students. All subjects were characterized by good physical fitness and did not participate in any structured sports training during the experiment. The sample were randomly assigned to three groups of fifteen participants (Table 1). The mean values of age, body mass, and body height were not statistically significantly different between the groups. The criterion for dividing into groups was the type of exercise performed. The G0 group performed continuous exercise of 60 repetitions without rest intervals, lasting about 180 s (Table 1). The task of those assigned to the G45 group was also to perform 60 repetitions, but in 4 sets of 15 repetitions, each set lasting 45 s, with 45 s rest intervals between the sets. The work-to-rest ratio in the G45 group was therefore 1:1. The G90 group also performed 60 repetitions in 4 sets of 15 repetitions each, lasting 45 s, but with twice as long rests (90 s) between sets. In the G90 group, the work-to-rest ratio was 1:2.

**Table 1:** Groups characteristic (Av. ± SD).

Group	Exercise type	One series duration (s)	Rest interval	Age (years)	Body mass (kg)	Body height (cm)
G0	1 × 60 rep	174 ± 5	–	22 ± 2	78 ± 10	183 ± 4
G45	4 × 15 rep	45.8 ± 1.6	45 s	20 ± 1	78 ± 7	183 ± 6
G90	4 × 15 rep	46.2 ± 2.1	90 s	20 ± 1	76 ± 8	180 ± 4

### Workout

**Figure 5:**
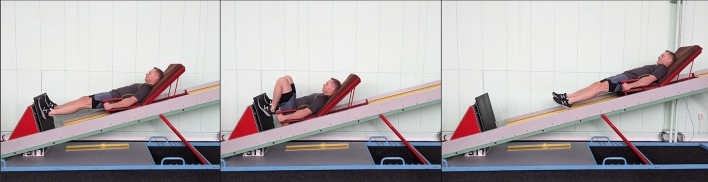
The sequence of the example bounce on the training machine.

Before beginning the measurements, the participants completed a standardized warm-up, during which they familiarized themselves with the exercise testing protocol. Exercises consisting of 60 rebounds on the incline plane training machine were used to maximize fatigue and monitor the biomechanical parameters of the exercise. The participant lay down on a special 44-kg cart and, rebounding with the lower limbs from the dynamometer platform, drove up an inclined plane with a 15° angle of the plane (Fig. 5). The participants were asked to perform each repetition at maximum intensity. Acting eccentrically, the muscles prevented the downward slide, and then, using concentric contraction, the participant performed a rebound, which in subsequent repetitions translated into work in the stretch–shortening cycle that is characteristic of plyometric exercises.

The rebounds from the dynamometric platform enable to obtaining of various biomechanical data of each repetition, such as speed, force, power, and work performed. In this study, power values relative to body mass were used to evaluate exercise intensity in the groups, while total mechanical work performed was used to evaluate exercise volume.

### Measurements

**Figure 6:**
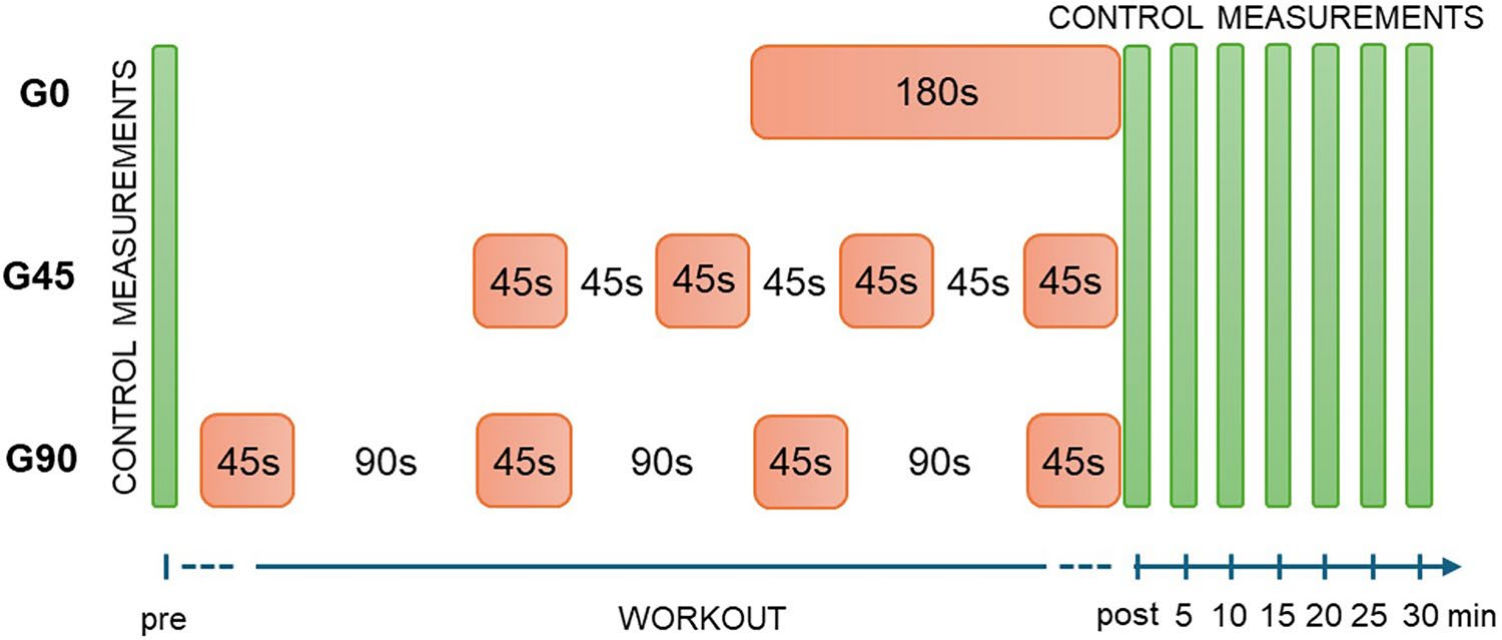
Time course of the experiment.

During the experiment, eight biomechanical and biochemical control measurements were taken: one pre workout, one immediately post, and six more every 5 min after completing the workout (Fig. 6).

To assess muscle fatigue after the exercise, maximum muscle torques under isometric conditions were measured for knee extensors (KE). The maximum torques of the antagonist muscles (knee flexors, KF), were also measured for comparison. The measurements were carried out on the TBK3-P stand for measuring moments of muscle torques in isometrics, which consisted of a seat with stabilization. The subject pressed his lower leg on a lever attached to the head of the GM6 torque meter equipped with a strain gauge torque transducer (sampling frequency 100 Hz, maximum measurement error < 0.5%, measuring range 2000 Nm, gain adjustment range 200× ÷ 1000×). Both measurement positions used a 90° angle at the knee joint. Measurements were taken before the exercise and at 5-min intervals afterwards for the following 30 min.

Blood for biochemical measurements was drawn from the earlobe before the exercise and at 5-min intervals after the exercise for the following 30 min. Capillary blood lactate (LA) levels were evaluated by spectrophotometric method, using a Lange mini-photometer. Evaluation of ammonia levels was performed using an Arkray PocketChem apparatus, and NH3 Ammonia test strip kit II.

### Statistical analyses

Based on the Shapiro–Wilk W-test, all data were found to have a normal distribution, so parametric statistical tests were used in further statistical analysis. A two-way ANOVA test for repeated measures (Group × Set) was used to assess the significance of changes for the power measurement during the exercise and a Group × TimePoint for the muscle torques, lactate, and ammonia measurements. In the case of significant ANOVA effects, detailed post-hoc comparisons between pairs of means were made using the Tukey test. Partial eta-squared (η^2^) was used as a measure of effect size. Correlation between quantitative characteristics was assessed using Pearson correlation. All analyses were performed using STATISTICA (TIBCO Software Inc. (2017). Statistica (data analysis software system), version 13. http://statistica.io). The level of *p* < 0.05 was set to evaluate the significance of the effects.

### Informed consent

The authors affirm that the experiment participant provided informed consent for publication of the image in Fig. 5 in an online open-access publication.

## Data Availability

The datasets generated during and/or analyzed during the current study are available from the corresponding author upon reasonable request.
